# Correction of PTEN mutations in glioblastoma cell lines via AAV-mediated gene editing

**DOI:** 10.1371/journal.pone.0176683

**Published:** 2017-05-02

**Authors:** Victoria K. Hill, Jung-Sik Kim, C. David James, Todd Waldman

**Affiliations:** 1 Georgetown University School of Medicine, Lombardi Comprehensive Cancer Center, Washington DC, United States of America; 2 Department of Neurological Surgery, Northwestern University Feinberg School of Medicine, Chicago, Illinois, United States of America; Virginia Commonwealth University, UNITED STATES

## Abstract

*PTEN* is among the most commonly mutated tumor suppressor genes in human cancer. However, studying the role of *PTEN* in the pathogenesis of cancer has been limited, in part, by the paucity of human cell-based isogenic systems that faithfully model *PTEN* loss. In an effort to remedy this problem, gene editing was used to correct an endogenous mutant allele of *PTEN* in two human glioblastoma multiforme (GBM) cell lines– 42MGBA and T98G. *PTEN* correction resulted in reduced cellular proliferation that was Akt-dependent in 42MGBA cells and Akt-independent in T98G cells. This is the first report of human cancer cell lines in which mutant *PTEN* has been corrected by gene editing. The isogenic sets of gene edited cell lines reported here will likely prove useful for further study of *PTEN* mutations in the pathogenesis of cancer, and for the discovery and validation of novel therapeutics targeting the PTEN pathway.

## Introduction

The *P**hosphatase and* *Ten**sin homolog* (*PTEN*) gene is a classical tumor suppressor gene targeted by loss-of-function mutations in a wide range of both familial and sporadic cancers [1,2]. Germline mutations in *PTEN* cause several phenotypically related familial tumor syndromes now collectively referred to as PTEN hamartoma tumor syndromes (PHTS) [3–6]. The most common of these is Cowden syndrome, which is characterized by numerous hamartomas, macrocephaly and an increased susceptibility to breast, endometrial, thyroid, and other cancers [7]. Somatic *PTEN* mutations are also common in sporadic cancers including uterine, glioblastoma multiforme, stomach and prostate, among many others [8–11]. A study across 12 different cancer types has shown *PTEN* to be the third most commonly mutated gene after *TP53* and *PIK3CA* [12].

PTEN is a 403 amino acid protein that functions as a dual protein and lipid phosphatase. Protein phosphatase activity has been observed against tyrosine-, serine- and threonine-phosphorylated proteins [13]. PTEN lipid phosphatase activity mostly functions to convert phosphatidylinositol 3,4,5-trisphosphate (PIP_3_) to phosphatidylinositol 4,5-diphosphate (PIP_2_) although PTEN also demonstrates activity against other phosphoinositides [14–16]. The lipid phosphatase activity of PTEN makes it a negative regulator of PI3K signaling and is considered essential for its tumor suppressor properties. Cellular accumulation of PIP_3_ through PI3K activity or PTEN inactivity results in recruitment of PDK1 to the cell membrane and subsequent phosphorylation and activation of Akt at T308 by PDK1 and S473 by mTORC2 [17–19]. Active Akt signaling then exerts numerous cellular effects including cell survival, cell cycle regulation, glycogen synthesis and cell growth [20].

In glioblastoma multiforme (GBM), alterations in the *PTEN* or *PI3K* genes are present in ~60% of all tumors [9,21,22] making it one of the most frequently altered pathways in this disease. Due to the many documented roles of Akt signaling, overexpression experiments are often not nuanced enough to identify the most important signaling events in a given cancer. Furthermore, *PTEN* knockout mice do not develop brain tumors [23–25], making it challenging to use genetically modified mice to study the role of *PTEN* inactivation in the pathogenesis of GBM. To create a more nuanced cell line model of *PTEN* in GBM, we have used human somatic cell gene targeting to correct a mutant allele of *PTEN* in two GBM cell lines. Analysis of these cell lines has revealed a striking proliferation phenotype in both sets of cells but conflicting roles for Akt involvement.

## Materials and methods

### Cell culture

42MGBA and T98G cells were obtained from DSMZ and ATCC, respectively. Cells were cultured in DMEM (Life Technologies) containing 10% fetal bovine serum (Sigma) and 1% penicillin/streptomycin (Life Technologies) at 37°C in 5% CO_2_.

### Human somatic cell gene targeting

An AAV-based gene editing vector for correction of tumor-derived mutations in exon 2 was designed. The left homology arm (LHA; ~1 kb) contains wild-type genomic sequence corresponding to intron 1, exon 2, and the first 200 nucleotides of intron 2. The right homology arm (RHA; ~1 kb) contains wild-type genomic sequence corresponding to intron 2. Homology arms were synthesized by Genscript and cloned into pAAV-SEPT, an AAV-based gene editing acceptor vector we previously reported in which polylinkers for the cloning of LHAs and RHAs flank a promoterless splice-acceptor-IRES-neo^R^ gene [26].

This *PTEN* gene editing vector was then packaged into AAV-2 virions (specifically, rAAV2/2) by co-transfection into HEK293T cells with helper plasmids pAAC-RC and pHELPER using X-tremeGENE 9 (Roche Diagnostics) according to manufacturers’ instructions. Two days after transfection, media was aspirated and cell monolayers were scraped into 1 mL PBS and subjected to four cycles of freeze/thaw. The lysate was then clarified by centrifugation at 12,000 rpm for 10 min in a benchtop microfuge to remove cell debris, and the virus-containing supernatant was aliquoted and stored at −80°C.

42MGBA and T98G recipient cells were then transduced with 200μl virus overnight in a T25 flask and plated out at limiting dilution into 96-well plates in 0.5mg/ml G418 containing media. Genomic DNA was extracted from G418 resistant colonies and tested for the presence of homologous integration of the targeting vector using a primer pair specific for the targeted allele. In 42MGBA cells, 7/100 colonies tested had undergone homologous recombination, three of which had undergone correction (in the other four, recombination occurred between the neo^R^ gene and the mutation, resulting in recombination without correction). In T98G cells, 12/200 clones tested had undergone homologous recombination, nine of which had undergone correction. DNA sequencing confirmed that a single mutant allele of *PTEN* was corrected in each of the two cell lines. Cells confirmed to have undergone mutation correction were expanded, infected with cre-expressing adenovirus overnight, and plated at limiting dilution into 96-well plates. Single colonies were expanded and tested for G418 sensitivity. Sensitive cells were expanded and tested for re-expression of PTEN protein (42MGBA) by Western blot or wild-type PTEN mRNA (T98G) by cDNA sequencing.

### cDNA sequencing

Total RNA was extracted using a Qiagen RNeasy mini kit according to manufacturers’ instructions. Immediately prior to cDNA synthesis, RNA was treated with DNAse I (Life Technologies) according to manufacturers’ instructions. cDNA was synthesized and subsequently amplified by PCR in one step using the SuperScript III one-step RT-PCR system (Life Technologies) and the following primers: forward (5’- CCCAGACATGACAGCCATC-3’); reverse (5’- TCTAGCTGTGGTGGGTTATGG-3’). cDNA sequencing was performed using the above primers and BigDye Terminator v3.1 Cycle Sequencing Kit (Applied Biosystems) according to manufacturers’ instructions.

### Western blot

Protein lysates were prepared in RIPA buffer (50mM Tris-HCL pH7.5; 150mM NaCl; 1% NP-40; 0.5% sodium deoxycholate; 0.1% SDS) containing complete mini protease inhibitors (Roche Diagnostics) and phosSTOP phosphorylation inhibitors (Roche Diagnostics). Western blots were performed using 4–12% NuPAGE Bis-Tris gels (Thermo Fisher Scientific) and run in 1xMOPS buffer. Proteins were transferred to PVDF membranes which were then probed with the following antibodies: PTEN clone 6H2.1 (Millipore #04–035). Akt (Cell Signaling #4691). Phospho-Akt S473 (Cell Signaling #4060). Phospho-Akt T308 (Cell signaling #2965). Tubulin alpha clone DM1A (NeoMarkers MS-581-P1).

### Cellular proliferation

Cells were plated into 96-well plates at either 500 or 750 cells per well in either normal serum conditions (10% FBS) or low serum conditions (1% FBS), and cell density was measured every 48 hours for 7 days using CellTiter-Glo (Promega) according to manufacturers’ instructions. When incubated in the presence of MK-2206, cells were plated as described and 24 hours later MK-2206 containing media or vehicle alone (DMSO) was added to the cells to a final concentration of 0.1 μM, 1.0 μM or 10 μM MK-2206.

## Results

### Gene editing to correct *PTEN* mutations in GBM cell lines

AAV-mediated gene editing was used to correct the endogenous, naturally-occurring *PTEN* mutations in two human GBM cell lines, 42MGBA and T98G. 42MGBA cells harbor a splice site mutation (c.164+1_164+2insG) in the exon 2 splice donor, resulting in aberrant splicing and the complete absence of PTEN expression [27]. T98G cells have a missense mutation in exon 2 of *PTEN* (c.125T>G) resulting in a leucine to arginine change at amino acid 42 (p.L42R), which is in the critical phosphatase domain of the protein [28].

To correct the mutations in 42MGBA and T98G cells, an AAV-based gene editing vector was designed and created, with two homology arms composed of wild-type *PTEN* genomic sequence flanking a FLOXed, promoterless IRES-neo^R^ gene (Fig 1a). Since the mutations in 42MGBA and T98G cells are both in exon 2, it was possible to use the same gene editing vector to correct the mutations in both cell lines. Next, transient stocks of AAV virus were generated and used to transduce 42MGBA and T98G cells. After infection, cells were plated at limiting dilution in G418-containing media, as described in detail in Materials and Methods. After several weeks of G418 selection and clonal growth, colonies were expanded, genomic DNA prepared, and tested by PCR for the presence of homologous integration of the KI vector. DNA was sequenced from parental cells and gene edited derivatives to confirm the presence of WT PTEN sequence (Fig 1b). Gene editing was heterozygous in both cell lines. Targeted clones were then transduced with cre-adenovirus to remove the neomycin resistance gene, restoring the now corrected *PTEN* allele to its otherwise original configuration (Fig 1a).

**Fig 1:**
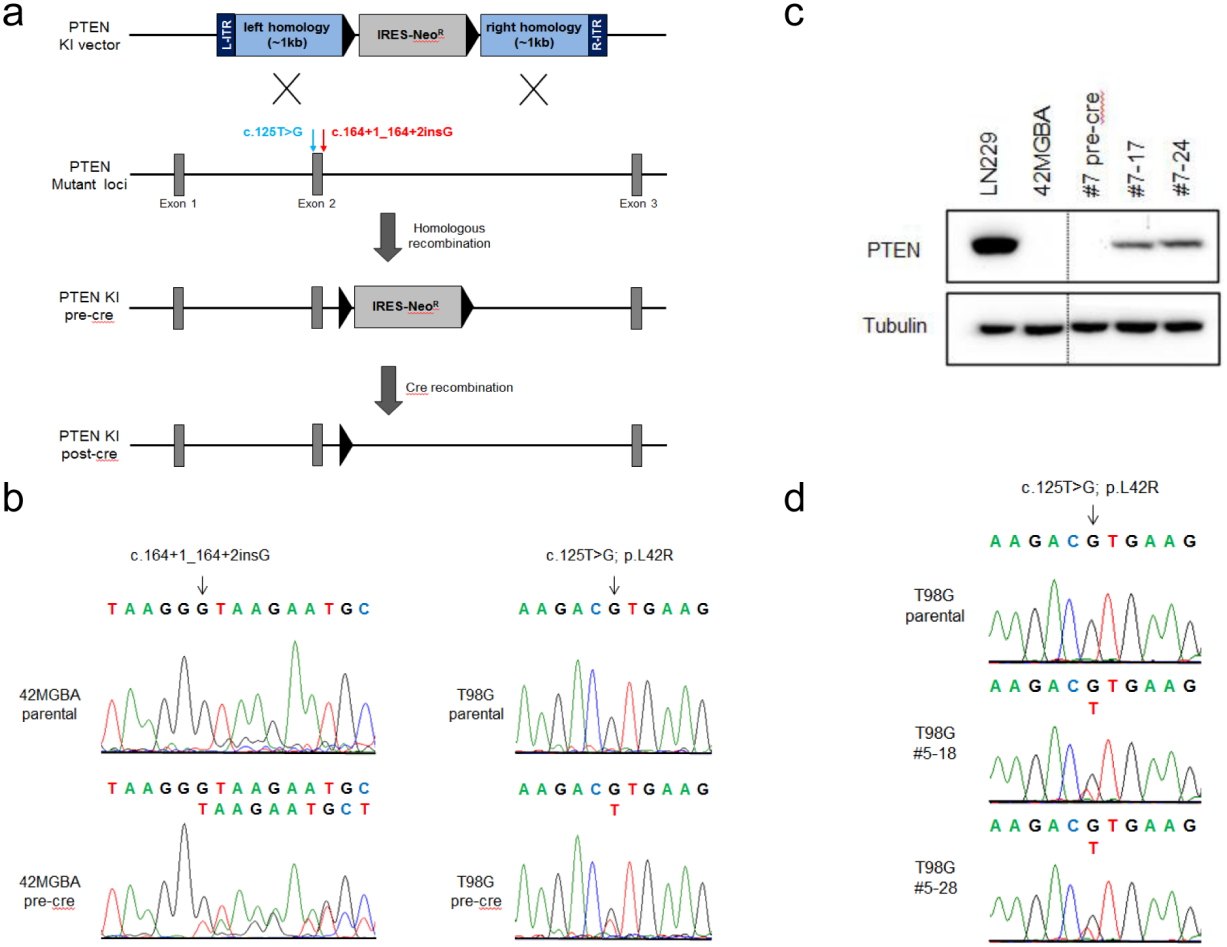
Generation of *PTEN* corrected GBM cell lines. (a) Schematic of the AAV-based gene editing approach utilized to correct *PTEN* mutations in 42MGBA and T98G cells. T98G mutation shown in blue text and 42MGBA mutation shown in red text. L-ITR, Left Inverted Terminal Repeat; R-ITR, Right Inverted Terminal Repeat; IRES-Neo^R^, Internal Ribosomal Entry Site-Neomycin resistance gene; KI, knock-in. (b) Sequencing of parental and gene edited 42MGBA and T98G cells demonstrates the homozygous nature of the endogenous mutation in parental cells, and heterozygous gene correction in gene edited derivatives. (c) Western blot for PTEN and tubulin expression with a positive control wild-type *PTEN* cell line (LN229), 42MGBA parental cells, pre-cre KI clone #7, and two independently-derived post-cre clones (#7–17, #7–24). (d) Sequencing of T98G *PTEN* cDNA demonstrates that both mutant and WT *PTEN* mRNA is expressed in two PTEN corrected clones (#5–18, #5–28).

In the case of 42MGBA cells (which completely lack expression of PTEN), Western blot was performed on parental cells and pre-cre and post-cre gene corrected clones, confirming that gene correction followed by cre recombination led to the re-expression of endogenous wild-type PTEN (Fig 1c). In the case of T98G cells (which express normal levels of mutant PTEN protein), RT-PCR was performed on RNA purified from parental cells and post-cre gene corrected clones and then sequenced, confirming that gene correction resulted in expression of wild-type *PTEN* mRNA (Fig 1d).

### Suppression of cellular proliferation after *PTEN* gene correction in 42MGBA and T98G cells

To assess the biological effects of *PTEN* correction, the CellTiter-Glo assay was used to measure the proliferation of 42MGBA and T98G parental cells and PTEN gene-corrected derivatives. Correction of *PTEN* in both 42MGBA and T98G cells resulted in substantially attenuated cellular proliferation (Fig 2a and 2b), as expected after re-expression of a wild-type tumor suppressor gene. These results confirm that *PTEN* gene correction has the expected biological effects in both GBM cell lines studied.

**Fig 2:**
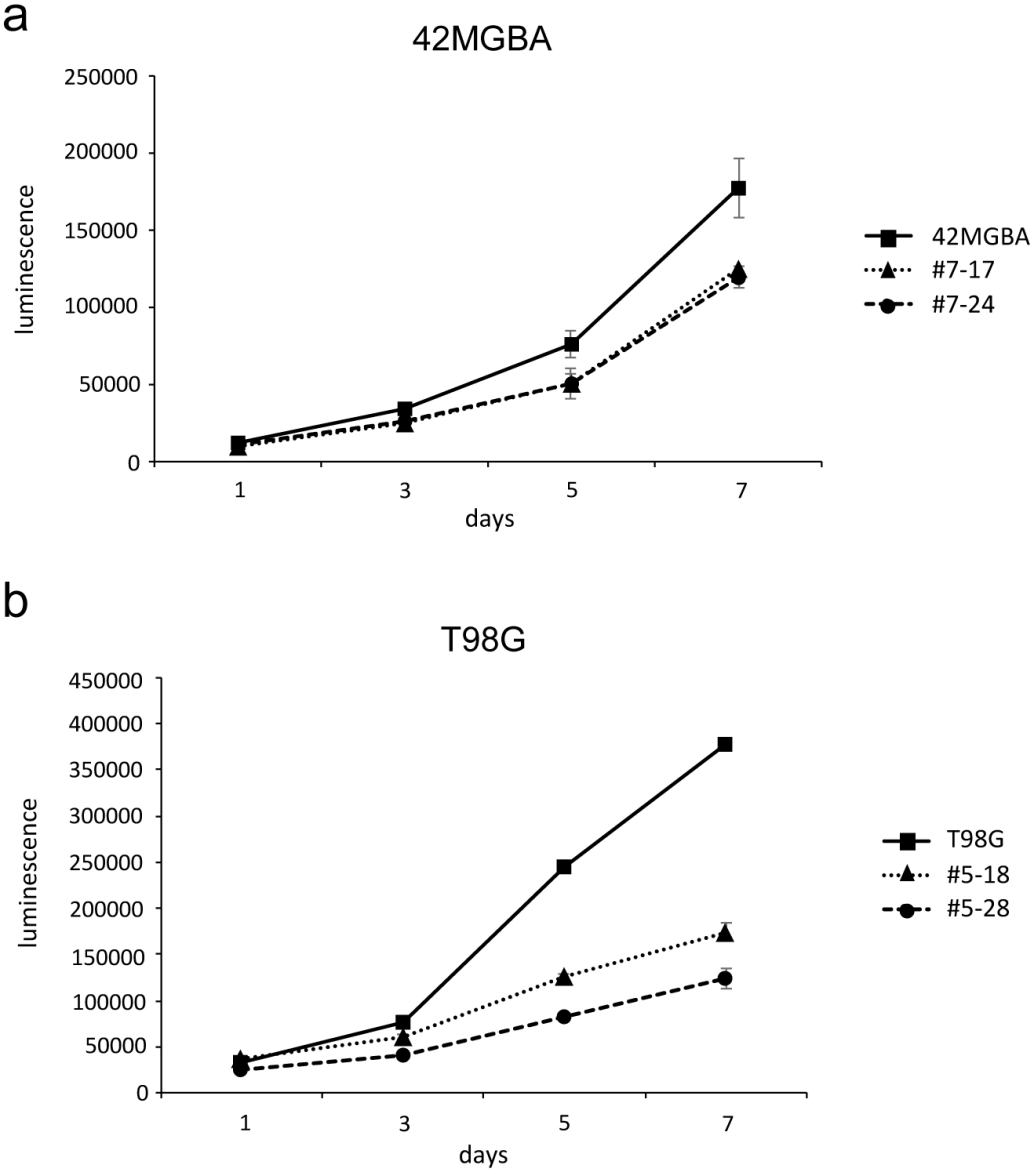
Anti-proliferative effects of *PTEN* correction. (a) The proliferation of isogenic sets of 42MGBA cells and PTEN corrected derivatives (#7–17, #7–24) were measured using the CellTiter-Glo assay (Promega). (b) Same as (a) for T98G parental cells and two independently-derived *PTEN* corrected clones (#5–18, #5–28). All cells were cultured in 10% serum conditions. 42MGBA cells were plated at 500 cells per well and T98G cells were plated at 750 cells per well.

### Modulation of Akt signaling by *PTEN* correction in 42MGBA cells but not T98G cells

Having confirmed that correction of *PTEN* was phenotypically meaningful in both cell lines, we next tested the effect(s) of *PTEN* correction on Akt signaling in 42MGBA and T98G cells. To do this, Western blots were performed on protein lysates derived from isogenic sets of parental cells and PTEN-corrected derivatives using pan-specific and phospho-specific antibodies for Akt. Correction of *PTEN* in 42MGBA cells resulted in the expected downregulation of Akt signaling, as demonstrated by a reduction in levels of phosphorylated Akt (S473 and T308) (Fig 3a and 3b). This effect was particularly prominent when the cells were cultured in 10% serum conditions (Fig 3a). In contrast, correction of the L42R missense mutation in T98G cells had no consistent effect on the phosphorylation of Akt, regardless of serum conditions (Fig 3c and 3d). These results suggest that the effects of *PTEN* correction on cellular proliferation in T98G cells is via an effect on pathway(s) other than the Akt signaling pathway.

**Fig 3:**
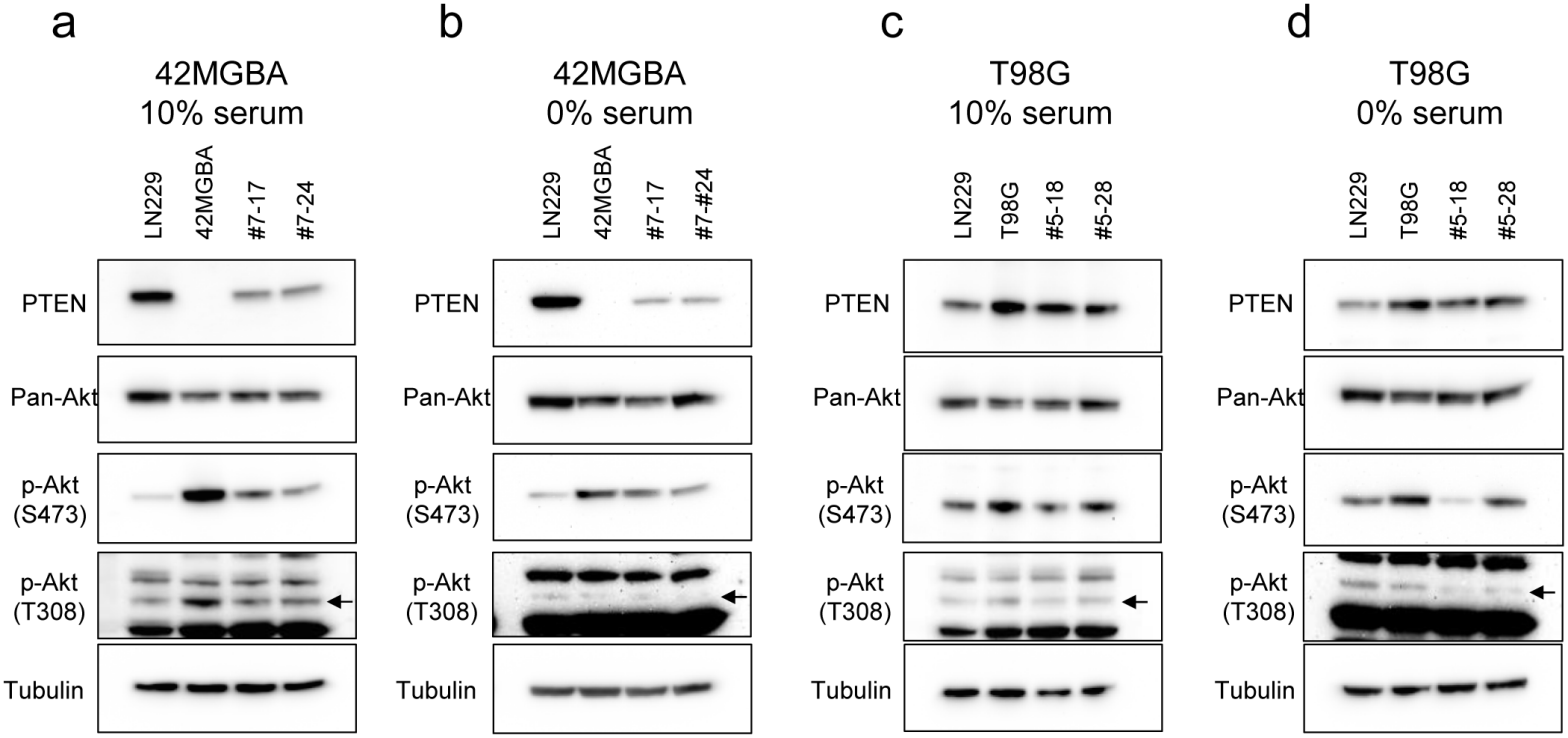
Akt phosphorylation in *PTEN* corrected 42MGBA and T98G cells. Western blot results are shown for PTEN, pan-Akt, p-Akt (S473), p-Akt (T308) and tubulin in LN299 *PTEN* wild-type control cells, 42MGBA cells, and two independently-derived *PTEN* corrected derivatives (#7–17 and #7–24) cultured in either 10% serum (a) or serum starved for 12 hours (b). Similar blots are shown in panels (c) and (d) for parental T98G cells and two independently-derived *PTEN* corrected clones (#5–18, #5–28) in 10% serum and serum starved for 12 hours, respectively.

### Phenotypic effects of pharmacological inhibition of Akt in 42MGBA and T98G cells

We next examined the effect of pharmacological inhibition of Akt on the proliferation of parental 42MGBA and T98G cells and their *PTEN* gene corrected derivatives. Initially, parental 42MGBA and T98G cells were treated with MK2206, an allosteric inhibitor of all three Akt isoforms, and Western blot and cellular proliferation assays were performed. As shown in Fig 4a, treatment of both cell lines with 1 μM MK2206 resulted in the virtual elimination of phosphorylated Akt, as expected for a potent pan-Akt inhibitor. At this concentration, MK2206 treatment had an anti-proliferative effect on 42MGBA cells but not on T98G cells (Fig 4b). Of note, a 10-fold higher concentration led to immediate cell death in all cell lines tested, consistent with previous studies indicating off-target effects for this drug at the highest concentrations [29].

**Fig 4:**
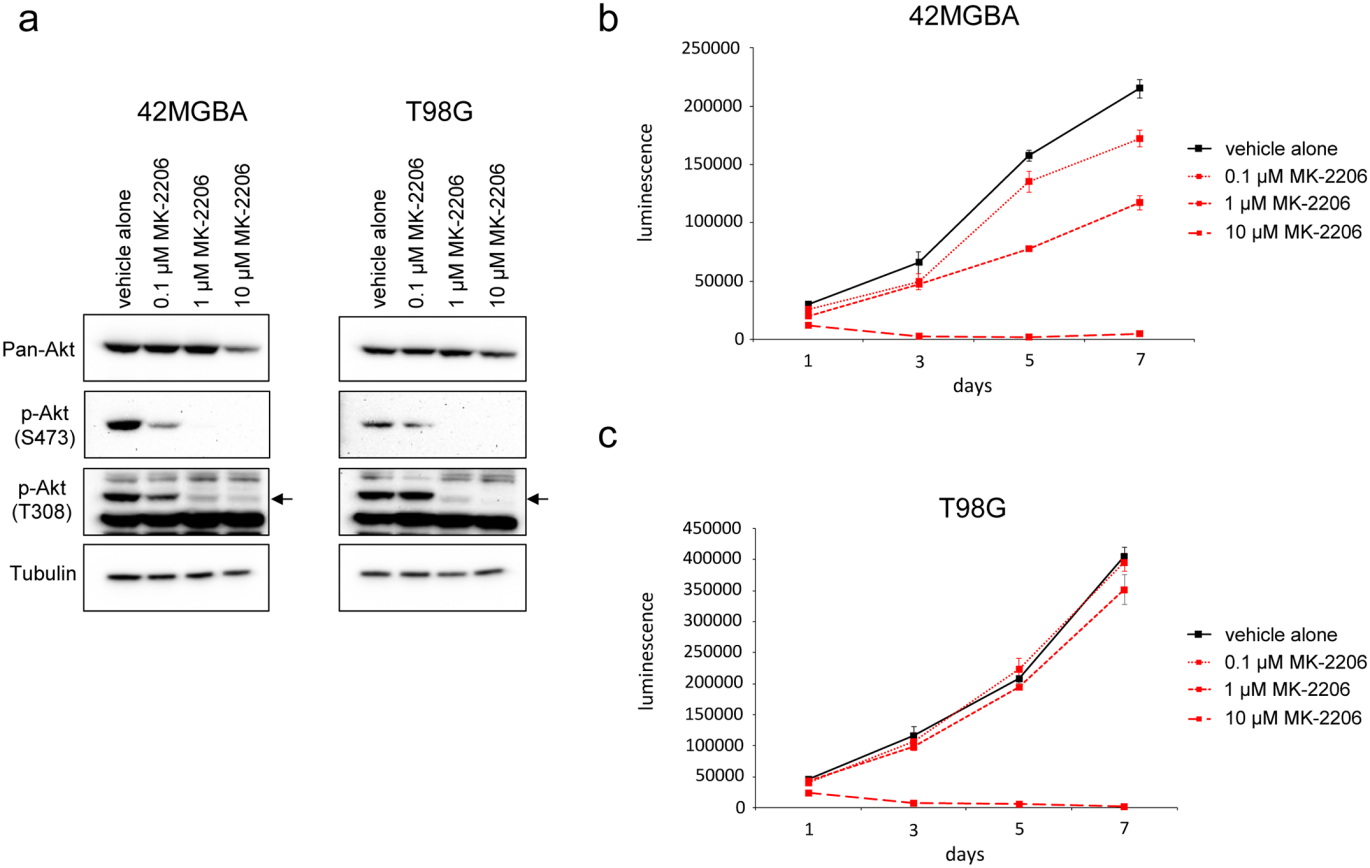
Pharmacological inhibition of Akt in 42MGBA and T98G cells. (a) Western blot results for pan-Akt, p-Akt (S473), p-Akt (T308), and tubulin in lysates from 42MGBA and T98G cells treated for 24 hours with either vehicle alone (DMSO) or increasing concentrations of MK-2206. CellTiter-Glo proliferation results over 7 days for parental 42MGBA cells (b) and T98G cells (c) cultured in either vehicle alone or increasing concentrations of MK-2206. Cells were plated at 500 cells per well.

Next, we expanded our analysis to include both 42MGBA and T98G parental cells and their isogenic derivatives with corrected alleles of *PTEN*. As shown in Fig 5a and 5b, while MK2206 had a potent anti-proliferative effect on *PTEN*-mutant 42MGBA cells, it had little effect on their *PTEN*-corrected derivatives, suggesting that the anti-proliferative effect of PTEN re-expression on 42MGBA cells was via an effect on Akt signaling. In contrast, treatment with 1.0 μM MK2206 had no adverse effect on either the proliferation of T98G parental cells or their *PTEN*-corrected derivatives (Fig 5c and 5d), despite its profound effect on Akt phosphorylation (Fig 4a). Taken together, these data suggest that activation of Akt signaling is required for the proliferation of 42MGBA cells but not T98G cells.

**Fig 5:**
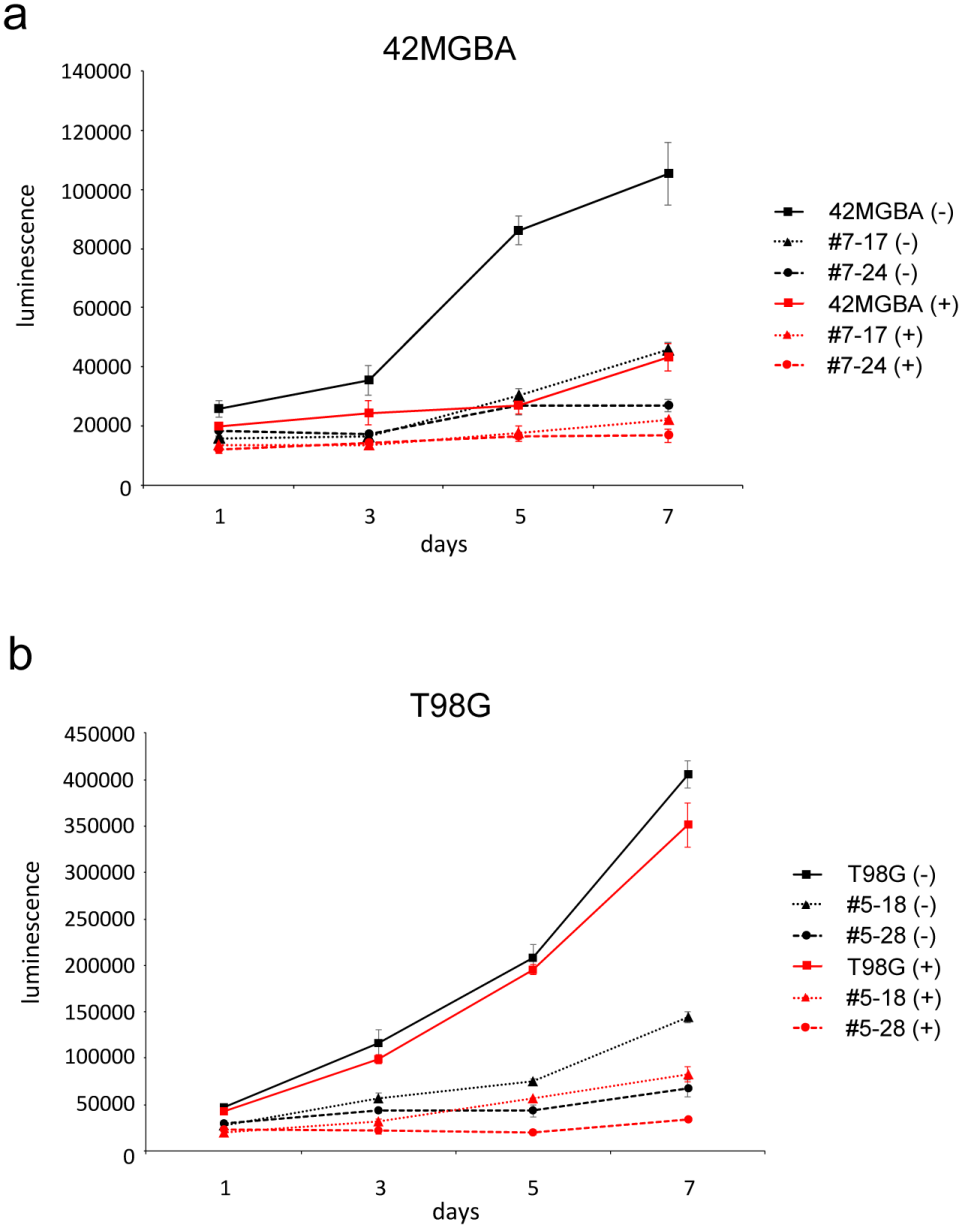
Pharmacological inhibition of Akt in *PTEN* corrected cells. (a) Cell proliferation results for 42MBGA cells and *PTEN* corrected derivatives (#7–17, #7–24) cultured in 1% serum either with (+) or without (-) MK-2206. Pharmacological inhibition of Akt inhibits cell proliferation in *PTEN* mutant 42MGBA cells but not in their isogenic *PTEN* corrected derivatives. (b) Cell proliferation results for T98G cells and *PTEN* corrected derivatives (#5–18, #5–28) cultured in 10% serum either with (+) or without (-). 500 cells were plated per well.

## Discussion

Here we report the creation and initial characterization of human GBM cells in which their endogenous mutant *PTEN* genes have been corrected by gene editing. Study of these isogenic sets of cells has revealed that correction of mutant *PTEN* leads to suppression of cellular proliferation which is Akt dependent in one cell line (42MGBA) and Akt independent in the other cell line (T98G).

The sets of cells reported herein are, to our knowledge, the first isogenic human cells in which a naturally-occurring mutant allele of *PTEN* has been corrected by gene editing. In fact, there are to our knowledge only two previous reports of isogenic sets of human cancer cells in which any mutant tumor suppressor has been corrected by gene editing–*TP53* and *STAG2* [30,31]. Correction of mutant tumor suppressor genes has only rarely been attempted because of the risk that successful correction will be incompatible with cellular proliferation, making it impossible to obtain gene-corrected clones. Instead, gene editing has more commonly been used to introduce inactivating mutations into wild-type alleles of tumor suppressor genes, since introducing an inactivating mutation into a wild-type tumor suppressor is predicted to enhance proliferation.

Despite this risk, targeted correction of an endogenous mutant tumor suppressor is the preferred experimental approach because the presence of a naturally-occurring mutation in a tumor suppressor gene indicates that the relevant cancer-causing pathway is specifically inactivated by that mutation. Therefore, gene correction will almost certainly result in pathway correction. In contrast, when knocking out a wild-type tumor suppressor, there is always the possibility that the pathway has already been inactivated by an unidentified genetic or epigenetic event in a different gene in the same pathway. In that case, knocking out the tumor suppressor will have little or no effect on the relevant cancer-causing pathway.

Study of the cells reported herein has demonstrated that *PTEN* mutations in human GBM have a pro-proliferative effect, since targeted correction of these mutations resulted in the inhibition of cellular proliferation. However, in only one of the cell lines (42MGBA) did *PTEN* correction lead to the expected modulation of Akt signaling, and only in that cell line did pharmacological inhibition of Akt lead to inhibition of proliferation. These data suggest that the PTEN-dependent proliferation effect in T98G cells is Akt-independent. Of note, our efforts to study the Akt-dependence of other proposed phenotypic effects of *PTEN* inactivation (invasion, growth as xenografts in immunodeficient mice) were unsuccessful because neither 42MGBA cells not T98G cells invades in standard invasion assays nor makes tumors when implanted subcutaneously in immunodeficient mice.

The *PTEN* mutation in T98G cells is a missense mutation (L42R) that has previously been described as having intact lipid phosphatase activity and behaving like wildtype PTEN in relation to Akt regulation [32,33]. Additionally, a more recent study has described L42R and 3 other missense mutations as having intact lipid phosphatase activity but a reduced ability to associate with the plasma membrane [34]. Nguyen et al also demonstrate improved PIP_3_-based regulation of Akt when an overexpressed version of L42R is artificially tethered to the membrane in cell lines exhibiting enhanced PIP_3_ signaling.

At present we do not know what PTEN-controlled pathway in T98G cells might be responsible for the effect on cellular proliferation. Previously reported Akt-independent mechanisms of *PTEN* tumor suppression include the regulation of a PTEN dependent cell size checkpoint [33], activation of JNK signaling pathways [35], activation of SRC signaling via PTEN protein phosphatase activity [36], phosphatase independent PTEN protein-protein interactions regulating the PKR-eIF2α phosphorylation pathway [37] and the oncogenic transformation of cells by MSP58 [38].

In summary, here we report the first human cancer cell lines in which mutant *PTEN* has been corrected by gene editing, and describe their initial characterization. Additional study of the mechanisms of *PTEN*-dependent growth suppression in T98G cells may shed light on the mechanisms of Akt-independent tumor suppression in GBM and other tumor types. Furthermore, the isogenic sets of cell lines described herein will likely prove useful for further study of *PTEN* mutations in the pathogenesis of GBM, and for the discovery and validation of novel therapeutics targeting the *PTEN* pathway.
